# Immersive Virtual Reality–Based Methods for Assessing Executive Functioning: Systematic Review

**DOI:** 10.2196/50282

**Published:** 2024-02-26

**Authors:** Rebecca Kirkham, Lars Kooijman, Lucy Albertella, Dan Myles, Murat Yücel, Kristian Rotaru

**Affiliations:** 1 Turner Institute for Brain and Mental Health School of Psychological Sciences and Monash Biomedical Imaging Facility Monash University Clayton Australia; 2 Institute for Intelligent Systems Research and Innovation Deakin University Geelong Australia; 3 Queensland Institute of Medical Research Berghofer Medical Research Institute Herston Australia; 4 Department of Psychiatry School of Clinical Sciences Monash University Clayton Australia; 5 Monash Business School Monash University Caufield Australia

**Keywords:** virtual reality, executive functioning, neuropsychological assessment, systematic review, psychometric properties, cybersickness, immersion, cognition

## Abstract

**Background:**

Neuropsychological assessments traditionally include tests of executive functioning (EF) because of its critical role in daily activities and link to mental disorders. Established traditional EF assessments, although robust, lack ecological validity and are limited to single cognitive processes. These methods, which are suitable for clinical populations, are less informative regarding EF in healthy individuals. With these limitations in mind, immersive virtual reality (VR)–based assessments of EF have garnered interest because of their potential to increase test sensitivity, ecological validity, and neuropsychological assessment accessibility.

**Objective:**

This systematic review aims to explore the literature on immersive VR assessments of EF focusing on (1) EF components being assessed, (2) how these assessments are validated, and (3) strategies for monitoring potential adverse (cybersickness) and beneficial (immersion) effects.

**Methods:**

EBSCOhost, Scopus, and Web of Science were searched in July 2022 using keywords that reflected the main themes of VR, neuropsychological tests, and EF. Articles had to be peer-reviewed manuscripts written in English and published after 2013 that detailed empirical, clinical, or proof-of-concept studies in which a virtual environment using a head-mounted display was used to assess EF in an adult population. A tabular synthesis method was used in which validation details from each study, including comparative assessments and scores, were systematically organized in a table. The results were summed and qualitatively analyzed to provide a comprehensive overview of the findings.

**Results:**

The search retrieved 555 unique articles, of which 19 (3.4%) met the inclusion criteria. The reviewed studies encompassed EF and associated higher-order cognitive functions such as inhibitory control, cognitive flexibility, working memory, planning, and attention. VR assessments commonly underwent validation against gold-standard traditional tasks. However, discrepancies were observed, with some studies lacking reported a priori planned correlations, omitting detailed descriptions of the EF constructs evaluated using the VR paradigms, and frequently reporting incomplete results. Notably, only 4 of the 19 (21%) studies evaluated cybersickness, and 5 of the 19 (26%) studies included user experience assessments.

**Conclusions:**

Although it acknowledges the potential of VR paradigms for assessing EF, the evidence has limitations. The methodological and psychometric properties of the included studies were inconsistently addressed, raising concerns about their validity and reliability. Infrequent monitoring of adverse effects such as cybersickness and considerable variability in sample sizes may limit interpretation and hinder psychometric evaluation. Several recommendations are proposed to improve the theory and practice of immersive VR assessments of EF. Future studies should explore the integration of biosensors with VR systems and the capabilities of VR in the context of spatial navigation assessments. Despite considerable promise, the systematic and validated implementation of VR assessments is essential for ensuring their practical utility in real-world applications.

## Introduction

### Background

Executive functioning (EF) has long been a focus of neuropsychological assessment because of the significant role it plays in everyday functioning. EF is an umbrella term for higher-order cognitive skills used to control and coordinate a wide range of mental processes and everyday behaviors [1-5], including “...mentally playing with ideas; taking the time to think before acting; meeting novel, unanticipated challenges; resisting temptations; and staying focused” [6]. Although a universally accepted definition of EF does not exist [5], there is agreement on the attributes of 3 core executive functions: inhibition, cognitive flexibility, and working memory [2,4,6]. These core executive functions support other higher-order executive functions such as reasoning, planning, and problem-solving [6-8]. As EF impairment has been linked to a variety of mental disorders [9], it is often considered a transdiagnostic risk factor [10].

Although traditional methods used to assess EF are popular [11,12] and well validated [13], they have been criticized for their lack of ecological validity [14,15]. Ecological validity, within the scope of this study, is defined as the “functional and predictive relationship between the person’s performance on a set of neuropsychological tests and the person’s behavior in a variety of real world settings” [16]. Specifically, we interpret ecological validity as comprising 2 principal components: representativeness—the degree to which a neuropsychological test mirrors the demands of a person’s daily living activities that it aims to evaluate [17], sometimes referred to as *verisimilitude* [18]—and generalizability—the extent to which test performance predicts an individual’s functioning in their daily living activities [17], also known as veridicality [18].

Traditional assessments tend to take a “construct-led” approach, with each test intended to isolate a single cognitive process in an abstract measure. This process of abstraction may limit the ecological validity of the measure by resulting in poor alignment between the test outcomes and real-world functioning. In turn, this produces a large amount of variance in EF that is unaccounted for by traditional tasks. For example, Chaytor et al [19] noted that traditional EF tests accounted for only 18% to 20% of the variance in the everyday executive ability of participants. This lack of explained variance may be attributed to the nature of the testing environment, the constructs assessed in isolation, the participant’s affective state, and the compensatory strategies available to the participant [19]. A related methodological issue, known as the “task impurity problem” [4,20], indicates that the score on an EF task usually reflects not only the systematic variance attributable to the specific aspect of EF targeted by that task but also the (1) systematic variance across multiple types of EF tasks, (2) systematic variance attributable to non-EF aspects of the task, and (3) nonsystematic (error) variance (see the study by Snyder et al [10] for a detailed review). Outside the testing environment, the process of making a decision or planning and eliciting goal-directed behavior in everyday life is often highly dynamic and influenced by numerous internal and external factors [13,14]. Therefore, an ecologically valid assessment tool will need to include relevant contextual, dynamic, and multidimensional features such as affect and physiological state, which traditional assessments cannot include.

Furthermore, although traditional EF assessment tools may be appropriate for clinical populations, they generate less information about functioning in relatively healthy individuals. For example, the Trail-Making Test (TMT) has routinely been administered as a neuropsychological assessment of driving performance. Although some studies have demonstrated a relationship between the two [21,22], others have shown no relationship [23], particularly in nonclinical populations [24,25]. Thus, although traditional tools are adequate for detecting more severe EF impairments, they are less effective in detecting subtle changes in EF and early decline. Increased test sensitivity to detect subtle intraindividual changes may enable better detection of the prodromal stages of cognitive decline. Early detection is important as it enables early intervention, which may in turn improve prognosis. For example, sensitive detection can identify the prodromal stages of Alzheimer disease in seemingly healthy individuals [26] and mild cognitive decline up to 12 years before clinical diagnosis [27]. Similarly, in a situation in which an individual requires a capacity assessment for an activity, traditional assessments may have limited utility for nonclinical populations. The triangulation of multiple data sources such as biosensors may increase sensitivity to better identify subtle changes in capacity.

To address the shortcomings of poor ecological validity and test sensitivity, research on psychological assessment has begun to investigate virtual reality (VR) technology as a means of providing a more naturalistic environment for evaluating EF in clinical neuropsychological assessments. VR enables the development of custom-designed simulated environments that can replicate real-life environments, potentially increasing its ecological validity through representativeness. In addition, VR could increase engagement [28,29], reduce test time, and better integrate data from biosensors with in-task events that facilitate assessment. The following sections will expand on these points and consider the importance of validating and assessing the reliability of VR for EF assessment.

### Ecological Validity and Representative Tests

There is an increasing emphasis on conducting EF assessments using tasks that resemble situations experienced in everyday life [30]. For example, the Multiple Errands Test (MET) [31] requires individuals to run errands in a real environment (eg, a shopping center). Empirical assessment of the MET has demonstrated its generalizability to daily functioning [32] and carer reports of daily functioning [33]. However, given that the MET is designed to be performed in real-life locations, it is impractical for routine administration by clinicians [34,35] and susceptible to the variable features of real-world environments that are outside experimental control. VR can mitigate these difficulties by maintaining the real-world environment without requiring travel while enabling fine-tuned control and uniform presentation of environmental characteristics [36]. Several studies [37-39] have investigated and developed platforms for this purpose, commonly known as the virtual MET.

### Engagement

VR has the potential to enhance individual engagement more effectively than traditional pencil-and-paper or computerized tasks by offering a fully immersive experience [40]. Recognized as a crucial aspect of cognitive assessment, engagement can be improved through gamification, thereby improving task performance [41]. “Serious games,” defined as games intended for a variety of serious purposes, such as training, learning, stimulation, or cognitive assessment [42], have been shown to be more engaging than nongamified tasks [43-45]. The unique immersive environment of VR captures increased attention, leading to reduced average response times and response time variability [46]. Notably, recent studies using electroencephalography (EEG)-based metrics have shown greater attention elicited in immersive VR paradigms than in 2D computerized assessments [46]. This heightened immersion and engagement in VR may enhance the reliability of the measures by capturing a more accurate representation of an individual’s best effort.

### Cybersickness

Despite their increased engagement, VR paradigms have the potential to induce cybersickness, which can threaten the validity of the paradigm. Cybersickness (ie, dizziness and vertigo) is akin to motion sickness but occurs in response to exposure to VR [47]. Previous research suggests that there is a negative relationship between cybersickness and cognitive abilities. For example, Nalivaiko et al [47] found that reaction times were moderately correlated (*r*=0.5; *P*=.006) with subjective ratings of nausea. Similarly, Sepich et al [48] found that participants’ accuracy on n-back task performance was weakly to moderately negatively correlated (*r*=−0.32; *P*=.002) with subjective cybersickness ratings. Therefore, there is reasonable concern that the potential benefits of engagement and ecological validity may be compromised if participants experience cybersickness.

### Validity, Reliability, and Sensitivity

Arguably, the biggest threat to the utility of VR platforms is that many studies do not document their validity and reliability. A meta-analysis showed that VR assessment tools are moderately sensitive to cognitive impairment across neurodevelopmental, mental health, and neurological disorders [49], demonstrating their promising application in clinical settings. Borgnis et al [50] reviewed the VR-based tools for EF assessment that are currently available, illustrating the plethora of platforms developing in this field. The works by Neguț et al [49] and Borgnis et al [50] highlight the utility of VR assessment tools to detect dysfunction and present the various tools in the literature created to investigate EF. Kim et al [51] provided an overview of the research trends using VR for neuropsychological tests and documented the cognitive functions assessed in each study. However, to the best of our knowledge, there is no overview or examination of the psychometric properties of these VR tools or how they are being evaluated.

Typically, novel measures and assessments are validated against current gold-standard tasks for concurrent validity [52]. Concurrent validity can be a reliable means of determining whether two assessments measure the same construct. However, concurrent validity can also occur when two tests contain the same problems, such as inaccurately measuring a particular construct in the same way. Sequentially, many VR tasks are being created from a “function-led” perspective but validated against “construct-led” tasks [53,54]. Given their different approaches, function-led and construct-led assessments should be validated in different ways or at least using several validation approaches. If function-led VR assessments improve upon the validity of current assessment methods, validation techniques may also need to go beyond comparisons with traditional assessments. For example, function-led VR assessments may be better validated against additional alternative methods, such as carer reports, real-life performance (eg, self-care, residence, transportation, and employment), and diagnostic trajectory [49] as opposed to validation through traditional (construct-led) assessment. Without incorporating tests of ecological validity, the potential advantages of VR may go unrecognized. Given the increasingly rapid development of VR neuropsychological assessments, it will be imperative to maintain high validation standards for these tools [55].

Establishing the reliability of novel VR EF assessments is also critical to the integrity of the outcomes. Reliability ensures that the measure yields consistent and repeatable results, a foundational element for test validity. Consequently, both reliability and validity ought to be evaluated for each measurement tool. Test-retest reliability, confirming consistency over time, should be accompanied by the interval between assessments and the correlation of the results. Internal consistency, typically measured using the Cronbach α, should also be reported for each target construct or domain of assessment. Importantly, for immersive VR EF assessments that evaluate multiple EF constructs, it is essential to report the α for each distinct construct rather than a collective coefficient. This is because the coefficient is intended to evaluate item consistency within a scale measuring a single construct; applying it across disparate constructs could be confusing and potentially misleading.

### Consistency of Terminology

Finally, to ensure psychometric precision and build on previous research, EF assessment paradigms must adopt consistent terminology for their target assessment constructs. The field of EF, although of significant interest to both researchers and clinicians, is marked by varied terminology for identical constructs. This issue, longstanding in EF research (see the study by Suchy [5]; for a review, see the study by Baggetta and Alexander [56]), presents challenges to VR in the EF assessment field. For instance, inconsistent terminology hinders the synthesis of research findings. Diverse labels such as “impulsivity” and “impulse control” might, upon examination, refer to the same underlying construct. Consequently, researchers aiming to extend the literature on “impulsivity” might overlook pertinent studies or exclude valuable references because of terminological discrepancies.

This literature review sought to examine and discuss the development of the VR tools used to assess EF with a specific focus on evaluating their psychometric properties. The studies selected for inclusion in this review were those that developed assessment tools for EF either holistically or in part. The aims of this review were to (1) determine the components of EF assessed using VR paradigms, (2) investigate the methods used to validate VR assessments, and (3) explore the frequency and efficacy of reporting participants’ immersion in and engagement with VR for EF assessment.

## Methods

Our review methodology followed the PRISMA (Preferred Reporting Items for Systematic Reviews and Meta-Analyses) statement [57]. In line with the literature, EF was defined as a set of executive functions, including inhibition, cognitive flexibility, and working memory [2,4,6], that support other higher-order executive functions, such as reasoning, planning, and problem-solving [6,8].

### Inclusion Criteria

Before conducting the literature search, the inclusion criteria were established. First, only peer-reviewed articles and conference proceedings (complete manuscripts) written in English would be included. Second, articles that detailed an empirical, clinical, or proof-of-concept study in which an immersive virtual environment (ie, using a head-mounted display, not a 2D computer screen) was reported to broadly investigate EF or higher-order cognition or that examined EF via a selection of one or more subconstructs (eg, inhibitory control and working memory) would be included. Finally, only articles with an adult participant population published after 2013 would be included. This temporal limit was based on the release date of the Oculus Rift Development Kit 1 as it was one of the first accessible products for public use of VR. Articles were identified through the EBSCOhost, Scopus, and Web of Science (WoS) citation databases. Scopus and WoS were chosen because of their prominence as citation databases [58]. To compensate for the bias toward engineering and natural science articles found through Scopus and WoS [59], EBSCOhost was searched for articles published in fields such as (clinical) psychology and medicine.

### Search Strategy

Keywords were developed by identifying 3 main components that the relevant literature should include. The 3 components were based on “Virtual Reality,” “Neuropsychological Tests,” and “Executive Function.” It was decided not to search for specific components of EF because of the lack of consensus in the field regarding its components. Rather, it was assumed that, if an article addressed EF or a component of EF, it would include “executive functioning” as a keyword in the title, abstract, or keywords. Other reviews looking broadly at VR paradigms have used similar search strategies [49].

In this study, key terms were developed by identifying synonyms for key components and concatenating them using the “AND” Boolean operator. The final keywords used for the search were as follows: ([“virtual” OR “artificial” OR “simulated”] AND [“realit*” OR “world” OR “environment”]) AND ([neuropsych* OR function* OR cognit*] AND [(executive AND function*) OR (high* AND order AND cognit*)] AND [assessment]).

Literature queries made through EBSCOhost were limited to the following databases: Academic Search Complete, AgeLine, AMED, Applied Science and Technology Source, CINAHL, E-Journals, Health Source Consumer and Nursing/Academic Edition, MEDLINE, Mental Measurements Yearbook, Psychology and Behavioral Sciences Collection, and all variations of the American Psychological Association databases. Furthermore, for the search, 3 topic fields (ie, title, abstract, and subject terms) were used to paste the keywords. The 3 topic fields were concatenated using the “OR” Boolean operator. Using the Scopus database, we implemented a basic search in the article title, abstract, or keywords using the keywords. No additional limitations were applied. Our search in WoS included all databases, and the advanced search method was used wherein keyword searches in the article title, abstract, and keyword topic fields were concatenated using the “OR” Boolean operator (ie, Title=(keywords) OR Abstract=(keywords) OR Keywords=(keywords)).

The results for each database were exported to Covidence systematic review software (Veritas Health Information) [60], which removed duplicates. All abstracts were screened independently by the first author and the senior author to determine whether the contents met the inclusion criteria. Full-text screening was also performed by the same authors. Any disagreement was discussed by the first (RK), second (LK), and senior (KR) authors.

### Data Extraction

The first and second authors completed the data extraction process by manually reviewing each manuscript; data items (see the following section) were recorded in a tabular format using Microsoft Excel (Microsoft Corp).

### Data Items and Synthesis

Demographic details, qualitative descriptions of the VR paradigm, user experience, cybersickness, immersion and engagement details, and comparative measures for validation purposes were extracted (Multimedia Appendix 1 [53-55,61-76]).

A qualitative evaluation of the studies included in the review was performed, meaning that the content of each manuscript was assessed based on the reported target constructs or constructs relevant to EF and the extent to which the reported VR task was related to the assessment of the target construct or constructs. To do this, studies were categorized based on the construct they targeted through their VR paradigm as reported by the authors of the respective articles. If multiple constructs were assessed in a single study, the study was included for each construct. No inferences were made about which cognitive construct or constructs was assessed based on the tasks that were reported in the manuscripts. For example, if an article indicated only that they used a VR version of the Stroop test (ST) but did not disclose which construct it assessed using this test, the study was not categorized under inhibitory control or cognitive flexibility but under the general factor “executive functioning.”

Next, it was indicated whether the articles explicitly or implicitly disclosed the way in which the comparative measures (such as particular metrics) were used to validate the VR paradigm. For instance, if the article directly stated a priori that they hypothesized a correlation between a VR task measuring inhibition and a validation task such as the ST, this was recognized as providing explicit validation for inhibition. Conversely, if an article indicated that participants completed the ST, which assessed inhibition and processing speed, and mentioned that the VR paradigm evaluated inhibition, it was considered to provide implicit validation for inhibition. Furthermore, traditional construct- and function-led assessments were identified from the text.

The (quantitative) results of the studies were screened to identify (1) the direction and strength of the relationship between traditional and VR assessments and (2) whether the results from all possible and a priori–defined comparisons were reported.

Finally, qualitative and quantitative tools used to evaluate beneficial and adverse effects of VR immersion were identified from the manuscripts and categorized in a tabulated format. The results of the studies were screened to identify whether they assessed the influence of the beneficial and adverse effects of VR immersion on task performance.

## Results

### Overview

**Figure 1:**
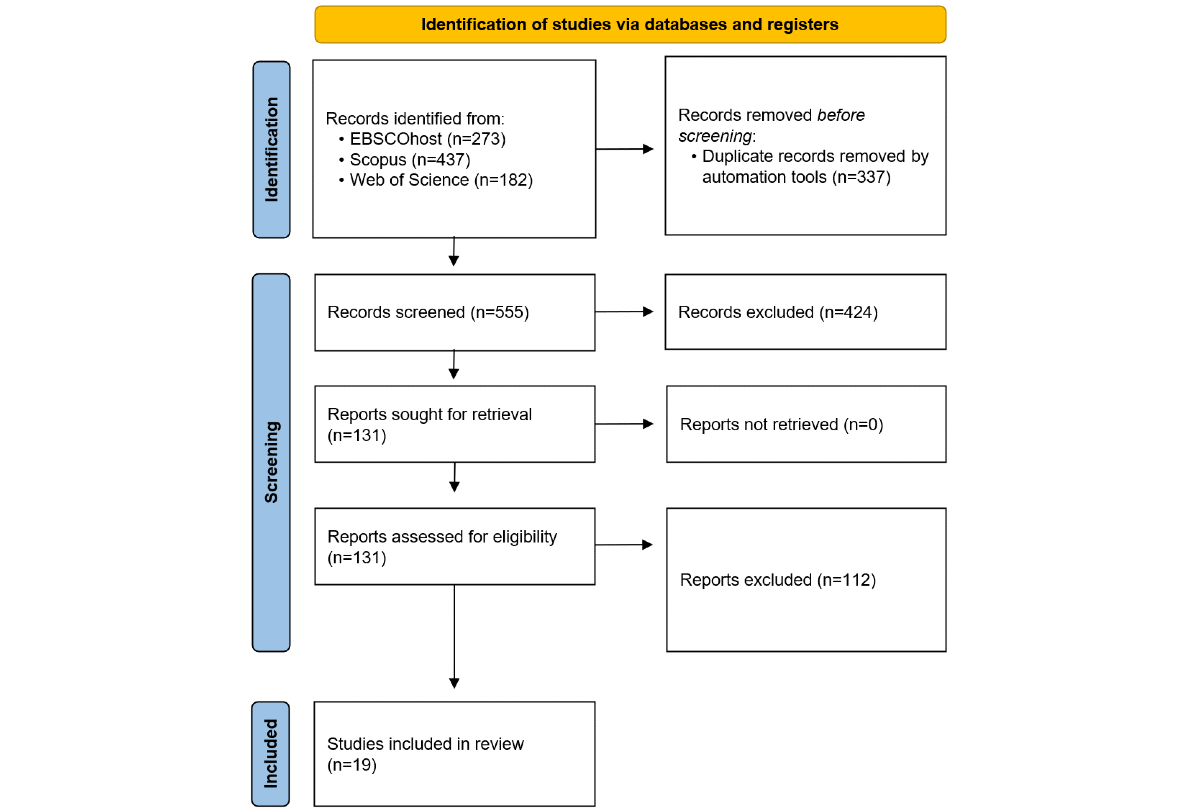
Systematic review process and results from literature searches in EBSCOhost, Scopus, and Web of Science databases.

Through WoS, EBSCOhost, and Scopus, 892 items were identified, from which the Covidence systematic review management platform [60] filtered 337 (37.8%) duplicates. A total of 555 unique articles remained, of which 424 (76.4%) were deemed irrelevant through abstract screening. The final 131 articles had their full texts screened, and 19 (14.5%) met the inclusion criteria. The systematic literature search process is shown in Figure 1.

### General EF

In total, 7 of the 19 (37%) of the reviewed studies assessed EF in general, meaning that the authors of these articles did not explicitly state which subconstruct of EF was targeted using the VR task. Table 1 shows which validation tasks were used in each study to measure EF.

**Table 1:** The validation tasks, authors, and total number of studies examining general executive functioning.

VR^a^ target construct and validation task			Validation		Authors		Studies examining the construct, n (%)	
**Executive functioning: general**								7 (37)
	D-KEFS^b^ [77] TMT-A^c^ and TMT-B^d^ ST^e^ Modified version of the SET^f^ HTT^g^ ZMT^h^	Implicit		Banville et al [61]^i^				
	ST TMT-A TMT-B	Implicit		Davison et al [62]^j^				
	TMT-B OTS^k^ CANTAB^l^ VFT^m^	Explicit		Miskowiak et al [63]				
	TMT-A TMT-B	Explicit		Pallavicini et al [64]				
	Groton Maze Learning Test (Cogstate)	Implicit		Porffy et al [65]				
	None specifically reported	N/A^n^		Tan et al [66]				
	None specifically reported	N/A		Tsai et al [67]				

^a^VR: virtual reality.

^b^D-KEFS: Delis-Kaplan Executive Function System.

^c^TMT-A: Trail-Making Test version A.

^d^TMT-B: Trail-Making Test version B.

^e^ST: Stroop test.

^f^SET: Six Elements Test.

^g^HTT: Tower of Hanoi test.

^h^ZMT: Zoo Map Test.

^i^The VR task was predominantly a sorting task for executive functioning assessment. The comparative assessments that validated this assessment were detailed under “executive function” broadly as the paper did not specify which components of the VR task the comparative tasks aimed to validate.

^j^The VR task was reported to assess executive functioning. The comparative assessments that validated this assessment were detailed under “executive function” broadly as the paper did not specify which components of the VR task the comparative tasks aimed to validate.

^k^OTS: One Touch Stockings of Cambridge.

^l^CANTAB: Cambridge Neuropsychological Test Automated Battery.

^m^VFT: verbal fluency test.

^n^N/A: not applicable.

Banville et al [61] immersed participants in a Virtual Multitasking Test (VMT), which was in principle designed to measure prospective memory and executive functions by having participants perform multiple tasks in a virtual apartment. However, this paper reported specifically on the task in which participants had to store groceries as fast as possible while also being attentive to other tasks, such as answering the phone or closing a window. Although the authors hypothesized that VMT scores would be correlated with neuropsychological assessments, such as mental flexibility, planning, and inhibition, it was not explicitly stated which metric of the VMT would be correlated with which neuropsychological assessment. Nonetheless, the authors identified that grocery storing time was correlated with the rule-break score on the Six Elements Test (*r*_19_=−0.49; *P*=.04; *P* value as reported in the manuscript). Furthermore, the number of errors in storing fruits and vegetables was found to correlate with the perseveration score on the Zoo Map Test (*r*_20_=0.53; *P*=.02; *P* value as reported in the manuscript) and reading speed during the second condition of the ST (*r*_20_=0.44; *P*=.05; *P* value as reported in the manuscript).

Davison et al [62] immersed participants in a parking simulator and a chemistry laboratory where they had to park a vehicle, sort chairs, or locate items. Before immersion, participants completed the ST and the TMT versions A (TMT-A) and B (TMT-B). The authors identified that the completion time of the second level (Kendall τ=−0.32; *P*=.01; *P* value as reported in manuscript) and the number of levels completed in the parking simulator (τ=0.43; *P*<.01; *P* value as reported in manuscript) were correlated with participants’ performance on the ST. In addition, the ST was correlated with seating arrangement metrics, such as time to place the first stool (τ=−0.33; *P*=.01; *P* value as reported in manuscript) and number of stools placed (τ=0.33; *P*=.02; *P* value as reported in manuscript), as well as with time to locate the first item in the chemistry laboratory (τ=−0.37; *P*=.01; *P* value as reported in manuscript). Correlations between the TMT-A or TMT-B and, for example, the number of completed parking levels (τ=−0.49; *P*<.01; *P* value as reported in the manuscript) or the number of items placed in the seating arrangement task in the chemistry laboratory (τ=−0.35; *P*=.01; *P* value as reported in the manuscript) were reported. However, reporting was limited to significant correlations only, and no a priori expectation of how performances on the VR and validation tasks were correlated was indicated in the study.

Miskowiak et al [63] assessed executive functions by letting participants complete the TMT-B, One Touch Stockings of Cambridge mean choices to correct, and verbal fluency test versions S and D. The performance on these tests was compared with participants’ performance on a cooking task in VR. The authors hypothesized that the number of cooking tasks that were correctly placed on a to-do list and the latency to solve the task would be VR-equivalent measures of EF. The authors found that VR performance was correlated (*r*_121_=0.26; *P*=.004) with EF, which consisted of a correlation between the average performance on the VR subtasks and the average performance on the validation tasks. The correlations between the individual performances on the VR and validation tasks were not reported in the manuscript.

Pallavicini et al [64] had participants play the Audioshield dance game, which the authors hypothesized could be closely related to EF constructs such as inhibition and working memory. However, the authors correlated participants’ performance on the Audioshield game with their performance on the TMT-A and TMT-B, which measure psychomotor speed (TMT-A) and mental flexibility (TMT-B). Nonetheless, the results showed that TMT performance was negatively correlated with Audioshield performance metrics.

Porffy et al [65] had participants complete VStore, where the 2 tasks measured EF, namely the “Find” task and the “Coffee” task. Specifically, participants had to find 12 items from a list they had previously memorized. In addition, participants had to order a hot drink from the coffee shop after finding, bagging, and paying for the 12 remembered items they had found in the store. Notably, the authors indicated that the 2 VR tasks also tapped into navigation (ie, "Find" task) and processing speed (ie, "Coffee" task). Furthermore, the Groton Maze Learning Test from Cogstate, which the participants completed before the VR task, was used to evaluate general EF. Nonetheless, through their regression analysis, the authors identified that the Groton Maze Learning Test was not a predictor for the "Find" task (*B*=0.024; SE 0.029; *P*=.11; *P* value as reported in the manuscript) or the "Coffee" task (*B*=−0.003; SE 0.051; *P*=.96; *P* value as reported in the manuscript).

Tan et al [66] had 100 participants complete 13 tasks in a virtual environment that were designed to measure 6 cognitive domains, such as EF and complex attention. Although differences in performance on VR tasks related to EF between age groups were found, no comparison was made with a traditional neuropsychological assessment of EF or any subconstructs of EF.

Tsai et al [67] immersed 2 participant groups in a virtual shopping environment: one group with mild cognitive impairment (MCI) and one control group. The VR tasks assessed participants’ memory, EF, and calculation by having them memorize a shopping list, search for the listed items in the shop, and subsequently pay for them. The authors trained machine learning models on features extracted from the VR tasks to predict whether participants had MCI or were healthy controls, which was achieved with high accuracy. Nonetheless, no neuropsychological assessment of EF was reported as a validation for the VR tasks.

### Targeted Constructs

The following subsections elaborate on the EF constructs and subconstructs addressed in the studies under review. A range of correlation coefficients were reported in these papers; however, because of the lack of uniformity in results reporting, these coefficients were omitted from the current synthesis. Typically, the papers reported only significant correlations between metrics without presenting all potential correlations. Furthermore, only 16% (3/19) of the studies specified an α level (ie, .05), with another 16% (3/19) of the studies indicating statistical significance at a *P* value of ≤.05. A total of 21% (4/19) of the studies did not indicate an α level but mentioned applying corrections for multiple comparisons, yet they did not detail the adjusted α level. In total, 5% (1/19) of the studies adopted Bayesian statistics using a Bayesian factor of >10 for statistical inference. Nonetheless, in the reviewed studies, it was not consistently clarified which VR tasks were validated against traditional tasks, hindering the construct validity of the various EF components. Consequently, drawing consistent conclusions on how EF constructs of subconstructs were evaluated was not feasible without inferring the nature of the tests and assessment paradigms.

### Core Executive Functions

#### Inhibition

Of the 3 “core” executive functions, 37% (7/19) of the studies included in our review investigated inhibitory control, interference control, or impulsivity either singly or combined. Table 2 details the respective validation tasks and target constructs of each of these studies. For example, Chicchi Giglioli et al [68] presented participants with 6 standardized tasks, 3 of which assessed inhibition (Table 2), before administering a serious game in which participants were required to perform tasks in outer space. In total, 10 of the 36 possible correlations between measures for the standardized tasks and the serious game tasks were reported as statistically significant and ranged from weak (0.20<*r*<0.39; relative *P* values indicated in the manuscript, eg, *P*<.05) to strong (0.60<*r*<0.79; relative *P* values indicated in the manuscript). For example, the latency metric of the dot-probe task (DPT) correlated positively (0.35<*r*<0.54; relative *P* values indicated) with the latency metric of the 3 VR tasks aimed at measuring inhibition, whereas no correlations were reported between the correct answer metric of the DPT and the correct answer metric of the 3 VR tasks aimed at measuring inhibition. None of the metrics from the ST correlated with those of the VR task (requiring participants to fight aliens); however, the correct answer and latency metrics of the ST correlated with those of the VR task (requiring participants to repair a valve).

**Table 2:** The validation tasks, authors, and total number of studies examining each construct.

VR^a^ target construct and validation task			Validation		Authors		Studies, n (%)	
**Inhibition or Inhibitory control**								6 (32)
	DPT^b^ GNG^c^ ST^d^	Implicit		Chicchi Giglioli et al [69]				
	DPT GNG ST	Explicit		Chicchi Giglioli et al [68]				
	GNG	Implicit		Marín-Morales et al [70]				
	CPT^e^	Implicit		Voinescu et al [71]^f^				
	None specifically reported	N/A^g^		Parsons and Carlew [72]				
	ST	Implicit		Parsons and Barnett [73]				
**Interference control**								3 (16)
	ST	Implicit		Marín-Morales et al [70]^h^				
	The CW-IT^i^ from the D-KEFS^j^ Automated neuropsychological assessment metrics ST	Implicit		Parsons and Carlew [72]				
	CW-IT from the D-KEFS	Implicit		Parsons and Barnett [73]				
**Impulsivity**								1 (5)
	None specifically reported	N/A		Chicchi Giglioli et al [68]				

^a^VR: virtual reality.

^b^DPT: dot-probe task.

^c^GNG: Go/No-Go.

^d^ST: Stroop test.

^e^CPT: continuous performance test.

^f^Some traditional tasks listed were included for divergent validity and, therefore, have been omitted from this table.

^g^N/A: not applicable.

^h^The VR task involved 42 VR mini-games that assessed various cognitive constructs. A total of 4 mini-games and their target constructs were documented and included in this table; however, the comparative assessments were not provided, and an extensive list of all 42 mini-games was not provided.

^i^CW-IT: Color-Word Interference Test.

^j^D-KEFS: Delis-Kaplan Executive Function System.

Similarly, Chicchi Giglioli et al [69] immersed participants in a virtual kitchen in which they had to cook different types of food. The activities were grouped into 4 subtasks of incremental difficulty where, in the third level, inhibition was assessed by determining whether the right dressing was added using a Go/No-Go (GNG)–type paradigm. The authors stated that the DPT, GNG, and ST were used as standard tasks to assess inhibition. The unspecified metric of “correct dressing” was shown to correlate well (*r*=0.527; *P*<.01; relative *P* value indicated in the manuscript) with the correct answer metric of the ST in one group, whereas in the second group, a moderate negative correlation (*r*=−0.486; *P*≤.05; relative *P* value indicated in the manuscript) was found between the execution time of the Tower of London task and the correct dressing metric. However, no other correlations between the VR task metric and those of the traditional assessments of inhibition were reported.

Marín-Morales et al [70] had participants complete neuropsychological assessments, including the GNG task, as well as 42 mini-games in VR. An undisclosed set of variables from the mini-games was used as predictors for measures of neuropsychological batteries. The mini-game predictor variables were fed into different machine learning algorithms. The authors highlighted that games related to inhibition produced worse results compared with other games but did not report any results on inhibition. The authors did find that mini-game features of planning and attention could predict GNG hit proportions and mean time with 80% and 94% accuracy, respectively.

Parsons and Carlew [72] had participants perform the ST in a virtual classroom as well as complete a computerized and paper-and-pencil version of the task. The authors found that participants’ performance was lower for color naming and word reading in the VR paradigm than in the paper-and-pencil version but interference performance was better in the VR paradigm than in the paper-and-pencil version. Similarly, Parsons and Barnett [73] had participants perform the ST in a virtual apartment as well as complete a computerized and paper-and-pencil version of the task. Here, the authors found that participants were more accurate in the ST in the paper-and-pencil version than in the VR paradigm.

Voinescu et al [71] immersed participants in a virtual aquarium where they had to perform a variety of tasks. For example, participants had to respond when they saw a fish that was different from a clown fish or heard a fish name different from surgeonfish. After the VR aquarium, participants completed a variety of computerized tasks, among them a continuous performance test (CPT), which was hypothesized to measure sustained attention and inhibition. The authors found weak to moderate (0.22<*r*<0.49; relative *P* values indicated, eg, *P*<.05) correlations between CPT measures and VR measures.

#### Working Memory

Working memory was investigated in 21% (4/19) of the studies [63,65,70,74]. Table 3 details the respective validation tasks and target constructs of each of these studies. The working memory component from the study by Marín-Morales et al [70] included a mini-game wherein participants had to recall the ingredients of a recipe seen before the mini-game and collect from a range of options only those ingredients found in the recipe. However, no correlations with neuropsychological tasks were presented. Miskowiak et al [63] compared their VR paradigm with a traditional task that assessed working memory. In this study, participants were instructed to plan and cook a meal in a virtual kitchen. Performance metrics, such as the number of drawers opened and the latency until the task was completed, were used to assess working memory and were correlated with metrics from traditional tasks such as the Wechsler Adult Intelligence Scale Letter-Number Sequencing. The authors reported a significant positive correlation (*r*_121_=0.31; *P*=.001) between the VR task metrics and the traditional task metrics that evaluated working memory.

**Table 3:** The validation tasks, authors, and total number of studies targeting working memory.

VR^a^ target construct and validation task		Validation	Authors	Studies, n (%)	
**Working memory**					4 (21)
	WAIS-IV^b^ The Working Memory Index (Digit Span and Arithmetic)	Implicit	Marín-Morales et al [70]^c^		
	WAIS-III^d^ LNS^e^ SWM^f^ CANTAB^g^ (error and strategy)	Explicit	Miskowiak et al [63]		
	1-back and 2-back test (Cogstate)	Implicit	Porffy et al [65]		
	None specifically reported	N/A^h^	Robitaille et al [74]^i^		

^a^VR: virtual reality.

^b^WAIS-IV: Wechsler Adult Intelligence Scale–IV.

^c^The VR task involved 42 VR mini-games that assessed various cognitive constructs. In total, 4 mini-games and their target constructs were documented and included in this table; however, the comparative assessments were not provided, and an extensive list of all 42 mini-games was not provided.

^d^WAIS-III: Wechsler Adult Intelligence Scale–III.

^e^LNS: Letter-Number Sequencing.

^f^SWM: Spatial Working Memory.

^g^CANTAB: Cambridge Neuropsychological Test Automated Battery.

^h^N/A: not applicable.

^i^Robitaille et al [74] used a VR paradigm with avatars to trial a dual-task walking protocol.

Porffy et al [65] asked participants to operate a virtual store in which the working memory component was assessed at the “Pay” step, where participants had to select and pay for their items at a self-checkout machine providing the exact amount. The authors specified that the reaction time on the 1-back task and the accuracy of performance on the 2-back task were metrics from traditional tasks used to assess working memory. Using linear regression, the authors found that performance on the 2-back task was negatively associated (*B*=−0.085; SE 0.042; *P*=.047) with participants’ performance on the “Pay” step.

Robitaille et al [74] assessed working memory during their simultaneous cognitive tasks, in which participants had to both recognize faces in windows that had been previously declared as “hostile” or “nonhostile” and complete a navigation task. However, no correlations between the traditional and VR tasks were reported.

#### Cognitive Flexibility

One study by Chicchi Giglioli et al [68] investigated cognitive flexibility (termed “cognitive shifting” in the paper) through 3 VR tasks. The authors specified that the TMT was used as a traditional task to assess cognitive flexibility as a comparator for the first VR task (CF1, cultivating food) and the Wisconsin Card Sorting Test was used as a traditional task to evaluate cognitive flexibility as a comparator for the other 2 VR tasks (CF2, growing plants, and CF3, fueling a turbine). The total time metric of the first VR task correlated positively with the total time of the TMT-B (*r*=0.396; *P*<.01; *P* value as reported in the manuscript), and multiple metrics of VR tasks 2 and 3 correlated with the performance metrics of the Wisconsin Card Sorting Test.

### Higher-Order Executive Functions: Planning

In total, 26% (5/19) of the studies [62,68,69,75,76] identified planning as a target construct in their VR paradigms. Table 4 details the respective validation tasks and target constructs of each of these studies. The VR environment created by Chicchi Giglioli et al [69] used a cooking task with 4 levels of difficulty. In the 3 more difficult levels, planning was required to complete the tasks as 2 burners were used. There was no clearly specified metric for the VR task that was used to evaluate planning, but the authors specified that the Tower of London task was used as a traditional assessment to evaluate planning. A variety of VR task metrics, such as total time to complete a difficulty level, were shown to correlate with various Tower of London task metrics.

**Table 4:** The validation tasks, authors, and total number of studies targeting planning.

VR^a^ target construct and validation task		Validation	Authors	Studies, n (%)	
**Planning**					5 (26)
	TOL-DX^b^	Implicit	Chicchi Giglioli et al [69]		
	TOL^c^	Explicit	Chicchi Giglioli et al [68]		
	None specifically reported	N/A^d^	Davison et al [62]^e^		
	The Key Search task from BADS^f^ [78]	Explicit	Kourtesis et al [76]		
	None specifically reported	N/A	Kourtesis and MacPherson [75]		

^a^VR: virtual reality.

^b^TOL-DX: Tower of London–Drexel test.

^c^TOL: Tower of London test.

^d^N/A: not applicable.

^e^The VR task was used to assess executive function. The comparative assessments that validated this assessment were detailed under “executive function” broadly as the paper did not specify which components of the VR task the comparative tasks aimed to validate.

^f^BADS: Behavioral Assessment of the Dysexecutive Syndrome.

In another study, Chicchi Giglioli et al [68] used a VR paradigm based on an outer-space environment. The paradigm contained 8 tasks, one of which assessed planning ability (task 7). The authors stated that the Tower of London task was the traditional assessment tool used to evaluate planning and explained that the total score, initial time, and execution time of the VR task were the outcome metrics. Moderate positive correlations were found between the execution time of the VR task and of the Tower of London task (*r*=0.463; *P*<.01; *P* value as reported in the manuscript) and between the initial time of the VR task and the total time of the Tower of London task (*r*=0.372; *P*<.05). Furthermore, the VR task correlated with some metrics of other traditional assessments used to assess planning ability, although these were not specified a priori.

Both the studies by Kourtesis et al [76] and Kourtesis and MacPherson [75] used the same VR environment based on a variety of everyday tasks. One task assessing planning ability required participants to draw their route around the city (eg, visiting the bakery, supermarket, and library and returning home) on a 3D board. Kourtesis et al [76] explained that the Key Search Test from the Behavioral Assessment of the Dysexecutive Syndrome was used as a traditional measure to assess planning and found a strong positive correlation between the traditional and VR tasks (*r*=0.80; Bayes factor=4.65 × 10^8^). Furthermore, Kourtesis and MacPherson [75] noted in their results that planning explained a substantial 12% (*P*=.03) of the variance in time-based prospective memory, which was required in 10 of 17 tasks.

Davison et al [62] assessed planning ability using a task involving the arrangement of a table and a chair. However, they did not explicitly mention the traditional task that was used to evaluate planning. Various correlations between the performance metrics of the VR task and the traditional task were reported. For example, the performance on the Stroop Color and Word Test was negatively correlated with the time participants took to place a blue chair in the seating arrangement task (Kendall τ=−0.39; *P*=.01; *P* value as reported in the manuscript).

### Other Domains

Several studies (14/19, 74%) examined domains of functioning that did not align with the EF definition used in this review. Broadly, these domains fell under the categories of memory, attention, processing, task performance, and a variety of other uncategorized subconstructs. As the literature [1,2,4,6] does not relate these broad domains to EF, they are not discussed further but are presented in Tables 5-6.

**Table 5:** The validation tasks, authors, and total number of studies targeting constructs classified as uncategorized.

VR^a^ target construct and validation task				Validation		Authors		Studies, n (%)	
**Memory**									11 (58)
	**Memory (general)**								1 (5)
		None specifically reported	N/A^b^		Tsai et al [67]				
	**Verbal memory and verbal learning**								2 (11)
		RAVLT^c^ subtests: total, immediate recall, delayed recall, and recognition	Explicit		Miskowiak et al [63]				
		International Shopping List Test (Cogstate; verbal learning)	Implicit		Porffy et al [65]				
	**Prospective memory**								4 (21)
		None specifically reported	N/A		Banville et al [61]^d^				
		CAMPROMPT^e^ [79]	Explicit		Kourtesis et al [76]^f^				
		None specifically reported	N/A		Kourtesis and MacPherson [75]				
		CVLT-II^g^ [80]	Implicit		Parsons and McMahan [53]				
	**Episodic memory**								3 (16)
		RBMT-III^h^ [81]	Explicit		Kourtesis et al [76]^f^				
		CVLT-II	Implicit		Parsons and McMahan [53]				
	**Immediate recognition**								2 (11)
		RBMT-III [81]	Explicit		Kourtesis et al [76]				
		None specifically reported	N/A		Kourtesis and MacPherson [75]				
	**Delayed recognition**								2 (11)
		RBMT-III [81]	Explicit		Kourtesis et al [76]^f^				
		None specifically reported	N/A		Kourtesis and MacPherson [75]				
**Attention**									13 (68)
	**General attention**								4 (21)
		DPT^i^ GNG^j^ ST^k^	Implicit		Chicchi Giglioli et al [69]				
		DPT GNG ST TMT-A^l^ TMT-B^m^	Explicit		Chicchi Giglioli et al [68]				
		DPT—selective attention GNG—sustained attention ST—selective attention TMT^n^—visual attention	Implicit		Marín-Morales et al [70]^o^				
		RVP^p^ CANTAB^q^ (accuracy and latency) RBANS-DS^r^	Explicit		Miskowiak et al [63]				
	**Divided attention**								2 (11)
		None specifically reported	N/A		Robitaille et al [74]^s^				
		CTT-B^t^ [75,82]	Explicit		Wilf et al [54]				
	**Complex attention**								1 (5)
		None specifically reported	N/A		Tan et al [66]				
	**Selective visual attention**								2 (11)
		The map task from the Test of Everyday Attention	Explicit		Kourtesis et al [76]^f^				
		None specifically reported	N/A		Kourtesis and MacPherson [75]				
	**Selective auditory attention**								2 (11)
		The Elevator Counting With Distraction task of the Test of Everyday Attention	Explicit		Kourtesis et al [76]^f^				
		None specifically reported	N/A		Kourtesis and MacPherson [75]				
	**Sustained visual attention**								1 (5)
		CTT-A^u^ [82]	Explicit		Wilf et al [54]				
	**Visuospatial attention**								2 (11)
		The Ruff 2 and 7 Selective Attention Test	Explicit		Kourtesis et al [76]^f^				
		None specifically reported	N/A		Kourtesis and MacPherson [75]				
	**Sustained attention**								1 (5)
		CPT^v^ [83]	Implicit		Voinescu et al [71]				
**Processing**									3 (16)
	**Processing speed**								3 (16)
		WAIS-IV^w^ Processing Speed Index (symbol search and coding)	Implicit		Marín-Morales et al [70]^o^				
		RBANS-CT^x^ TMT-A	Explicit		Miskowiak et al [63]				
		Detection task (Cogstate)	Implicit		Porffy et al [65]				
**Task performance**									4 (21)
	**Dual task**								1 (5)
		TMT-A TMT-B	Implicit		Chicchi Giglioli et al [69]				
	**Multitask**								3 (16)
		Modified version of the SET^y^	Implicit		Banville et al [61]^d^				
		CTT^z^ [82]	Explicit		Kourtesis et al [76]^f^				
		None specifically reported	N/A		Kourtesis and MacPherson [75]				

^a^VR: virtual reality.

^b^N/A: not applicable.

^c^RAVLT: Rey Auditory Verbal Learning Test.

^d^The VR task was predominantly a sorting task for executive function assessment. The comparative assessments that validated this assessment were detailed under “executive function” broadly as the paper did not specify which components of the VR task the comparative tasks aimed to validate.

^e^CAMPROMPT: Cambridge Prospective Memory Test.

^f^Kourtesis et al [76] explicitly broke episodic memory down into immediate and delayed recognition. However, we gathered these two constructs under episodic memory.

^g^CVLT-II: California Verbal Learning Test–Second Edition.

^h^RBMT-III: Rivermead Behavioral Memory Test–Third Edition.

^i^DPT: dot-probe task.

^j^GNG: Go/No-Go.

^k^ST: Stroop test.

^l^TMT-A: Trail-Making Test version A.

^m^TMT-B: Trail-Making Test version B.

^n^TMT: Trail-Making Test.

^o^The VR task involved 42 VR mini-games that assessed various cognitive constructs. In total, 4 mini-games and their target constructs were documented and included in this table; however, the comparative assessments were not provided, and an extensive list of all 42 mini-games was not provided.

^p^RVP: Rapid Visual Information Processing.

^q^CANTAB: Cambridge Neuropsychological Test Automated Battery.

^r^RBANS-DS: Repeatable Battery for the Assessment of Neuropsychological Status–Digit Span.

^s^Robitaille et al [74] used a VR paradigm with avatars to trial a dual-task walking protocol.

^t^CTT-B: Color Trails Test B.

^u^CTT-A: Color Trails Test A.

^v^CPT: continuous performance test.

^w^WAIS-IV: Wechsler Adult Intelligence Scale–IV.

^x^RBANS-CT: Repeatable Battery for the Assessment of Neuropsychological Status–Coding Test.

^y^SET: Six Elements Test.

^z^CTT: Color Trails Test.

**Table 6:** The validation tasks, authors, and total number of studies targeting constructs classified as uncategorized.

VR^a^ target construct and validation task				Validation		Authors		Studies, n (%)	
**Uncategorized^b^**									12 (63)
	**Visual perception**								1 (5)
		None specifically reported	N/A^c^		Marín-Morales et al [70]^d^				
	**Verbal learning**								2 (11)
		RAVLT^e^ subtests: total, immediate recall, delayed recall, and recognition	Explicit		Miskowiak et al [63]				
		International Shopping List Test (Cogstate)	Implicit		Porffy et al [65]				
	**Navigation**								2 (11)
		None specifically reported	N/A		Porffy et al [65]				
		None specifically reported	N/A		Robitaille et al [74]				
	**Associate learning**								1 (5)
		Continuous Paired Associate Learning Test (Cogstate)	Implicit		Porffy et al [65]				
	**Pattern recognition**								1 (5)
		Continuous Paired Associate Learning Test (Cogstate)	Implicit		Porffy et al [65]				
	**Perceptual motor**								1 (5)
		None specifically reported	N/A		Tan et al [66]				
	**Social cognition**								1 (5)
		None specifically reported	N/A		Tan et al [66]				
	**Learning and memory**								1 (5)
		None specifically reported	N/A		Tan et al [66]				
	**Language**								1 (5)
		None specifically reported	N/A		Tan et al [66]				
	**Calculation**								1 (5)
		None specifically reported	N/A		Tsai et al [67]				

^a^VR: virtual reality.

^b^Williams et al [55] replicated the Wisconsin Card Sorting Test and multitasking task but did not explicitly state the cognitive constructs that the VR task was assessing. For this reason, the paper has not been assigned a target construct.

^c^N/A: not applicable.

^d^The VR task involved 42 VR mini-games that assessed various cognitive constructs. In total, 4 mini-games and their target constructs were documented and included in this table; however, the comparative assessments were not provided, and an extensive list of all 42 mini-games was not provided.

^e^RAVLT: Rey Auditory Verbal Learning Test.

### Validity and Reliability

Tables 1-6 show details of the current validated comparator tasks against the novel VR tasks if they were explicitly provided by the authors. Where “None specifically reported” is stated, the authors of each paper did not identify or indicate a direct comparator. All but 2 studies (17/19, 89%) [72,73] set out to assess multiple constructs. In some cases, the subconstructs that were assessed were individually validated against existing validated tasks. In other cases, a suite of existing validated tasks was included in the analysis for correlation against a variety of subconstructs being assessed using the VR battery. In these cases, there was no validation at the construct level identified a priori. In 16% (3/19) of the studies, there was no reported validation of the VR paradigm.

Notably, only one study used real-life validation criteria in addition to construct-driven tests to present a validation of their VR paradigm. Specifically, Miskowiak et al [63] functionally assessed participants using the Functioning Assessment Short Test (FAST) and the brief University of California, San Diego, Performance-Based Skills Assessment (UPSA-B). Participants’ scores on these assessments were correlated with their performance on the test domains of the VR paradigm, called cognition assessment in VR (CAVIR). The authors identified that participants’ performance on the FAST was negatively associated (−0.17<*r*<−0.30; no exact or relative *P* values reported) with CAVIR test domains such as processing speed and working memory, whereas participants’ performance on the UPSA-B was positively associated with the CAVIR test working memory (*r*=0.40; *P* value not exactly or relatively reported) and cognition composite (*r*_68_=0.44; *P*<.001) domains. Moreover, the authors noted that lower global scores on traditional (ie, construct-led) neuropsychological tests were negatively associated with FAST scores (*r*_121_=−0.45; *P*<.001) and positively associated with UPSA-B scores (*r*_68_=0.52; *P*<.001), highlighting that lower CAVIR scores were associated with more functional disability, as indicated by the functional and traditional assessment tools.

The reliability of the VR paradigm was only assessed in 5% (1/19) of the studies. This was done by Kourtesis et al [76], who reported good internal reliability (Cronbach ⍺=.79) of their VR Everyday Assessment Lab (EAL) paradigm. However, this global internal consistency report did not provide a reliability estimate of the unique cognitive functions targeted by their VR EAL paradigm. Nonetheless, none of the reviewed studies included a test-retest analysis to highlight the reliability of their VR paradigm.

### Evaluation of User Experience, Cybersickness, Immersion, and Engagement

An overview of the measures used to evaluate participants’ experiences and well-being can be found in Multimedia Appendix 1 [53-55,61-76]. Of the 19 studies, 5 (26%) included user experience assessments. To measure participants’ virtual presence, experience, and well-being, the studies administered the Igroup Presence Questionnaire [61], Presence Questionnaire [63,71,74], or Slater-Usoh-Steed questionnaire [74]. To measure participants’ discomfort, the studies used the Simulator Sickness Questionnaire [61,71,74] or an adaption of it, the Virtual Reality Sickness Questionnaire [63]. To evaluate the usability of the virtual environment, the studies used the System Usability Scale [71]. To measure participants’ virtual experience and comfort, 11% (2/19) of the studies used the Virtual Reality Neuroscience Questionnaire [76].

Two studies (2/19, 11%) investigated whether system usability, virtual presence, or cybersickness affected participants’ task performance. For example, Porffy et al [65] measured participants’ technical familiarity and found that it explained between 10% and 42% of the variability in participants’ performance on the VStore outcomes "Recall", "Find", and "Select". Conversely, participants’ technical familiarity appeared to influence their performance on VStore. Kourtesis et al [76] used questionnaires to evaluate the quality of the VR paradigm, participants’ gaming experience, and the realism (verisimilitude) and pleasantness of the VR paradigm. The authors identified no relationship between VR experience, gaming experience, and performance on the VR EAL tasks.

Some papers (4/19, 21%) reported on cybersickness, presence, or usability scores but did not report an analysis of the relationship between task performance and measures evaluating the VR paradigm. For example, Banville et al [61] recorded participants’ sickness and virtual presence but did not report any test evaluating whether sickness or presence affected task performance. Similarly, Voinescu et al [71] obtained system usability ratings from participants; however, no test was reported wherein the effect of usability on task performance was assessed. Finally, Chicchi Giglioli et al [68] recorded participants’ use of technology but did not report an analysis between technology use and task performance.

Finally, some studies (2/19, 10%) evaluated participants’ experiences post hoc, although it was not disclosed whether any validated scales were used. For example, Davison et al [62] measured participants’ enjoyment of the VR tasks and their preference for either the VR tasks or the pencil-and-paper tasks. The authors found that younger participants rather than older ones preferred VR tasks over pencil-and-paper tasks. In addition, 11 out of 40 participants reported having experienced a mild degree of motion sickness. However, 58% (11/19) of the papers did not disclose any information about user experiences.

## Discussion

### Overview

The purpose of this review was to investigate the development and validation of VR assessment tools for EF. Specifically, we examined the components of EF that were assessed using VR, their validation processes, and whether immersion and cybersickness assessments were used. Although research in this domain is proliferating, the results of this review suggest that the process of development and validation varies considerably between studies.

### Components of EF Assessed Using VR Paradigms

#### Overview

The terminology used in the papers to describe EF constructs was inconsistent. For example, the most popular construct set assessed using VR comprised the inhibition processes. “Inhibitory control” encompasses the inhibition of goal-irrelevant stimuli, cognitions, and behavioral responses [6,84]. In total, two of the key components of inhibitory control are response inhibition and attentional inhibition [85]. Response inhibition was also termed “inhibition control,” “prepotent response inhibition,” and “motor inhibition,” whereas attentional inhibition was also termed “control of interference,” “interference control,” and “external interference control.” Although these terms are used in the literature [85], its readability and synthesis would be improved through agreement on a particular term for the same construct. In the same way, several studies (7/19, 37%) examined “EF” broadly without specifically detailing its components. In these studies, EF was validated using different measurement tools, suggesting that, across studies, EF was defined and used differently in each VR paradigm. As the constructs that these paradigms aimed to assess were not explicitly detailed, this poses a risk of hampering researchers wishing to build upon previous findings.

Furthermore, there was a broad range of constructs that were not commonly considered as EF domains but were reported as components of EF, making it difficult for future research to replicate the findings of undefined target constructs. For example, several papers (14/19, 74%) reported on verbal learning [63], associate learning pattern recognition [65], perceptual motor, social cognition, language [66], and calculation [67]. Although many of these components rely on EF domains or underpin those domains, they exist at various levels of abstraction. Thus, although the reviewed studies investigated components at different levels and used different languages, it is possible that they overlapped. For example, “organization” may be an umbrella term for a range of EF domains, each of which uses different terminology for the same concept, such as “cognitive flexibility,” “flexible updating,” and “working memory.” Although “organization” is not measured as a higher-order version of the subcomponents, it is difficult for the research that has examined cognitive flexibility and working memory to be extended. Thus, 2 studies assessing the same construct are not able to build on each other’s progress.

#### Recommendation: Establish a Coherent and Consistent Framework for EF Terminology

The Research Domain Criteria (RDoC) framework developed by the National Institute of Mental Health could serve as a framework to address this recommendation. The RDoC was originally created to consolidate the research conducted in various fields of mental health [86]. The framework categorizes cognition into 6 domains and encourages the investigation of these domains via different classes of variables, such as behavioral, physiological, and self-report data. This framework encourages a common language and organizes findings in such a way that researchers can identify gaps or discrepancies in the literature and contribute to the ongoing development of the field. This framework indicates the potential benefits of using a common language for research, although it is not necessarily the only option in this field. Alternatively, researchers could engage in a Delphi study to generate expert-informed consensus on the key constructs of EF that merit investigation using VR paradigms (eg, see the study by Yücel et al [87] for a Delphi study on neuropsychological assessment for addiction). Nonetheless, the emerging area of VR development for neuropsychological assessments would benefit from using the RDoC framework to coordinate the research process.

### Validation of VR for EF

#### Overview

Overall, there was limited reporting on the constructs that were assessed using VR paradigms and the associated validation outcome measures. In some papers, there was inadequate reporting of the constructs that the VR paradigm was intended to assess. In others, the same construct was assessed using a variety of traditional tasks. Furthermore, some VR paradigms were intended to replicate real life yet were validated against traditional tasks, none of which assessed ecological validity. In some studies, the correlations between the VR paradigm and the traditional tasks were incomplete. Finally, sample sizes varied considerably between studies, also affecting the evaluation of their psychometrics. These points are expanded upon in this section.

Several studies (5/19, 26%) examined EF as a broad category and then validated the paradigm against a variety of traditional tasks. However, some studies (3/19, 16%) detailed limited (or no) reporting of which aspect of the VR paradigm each traditional task was intended to validate. That is, no details were provided regarding which traditional task outcome measure corresponded to each component of EF within the VR paradigm. Traditional tasks, which often target one construct, were then correlated against seemingly all outcomes of the VR paradigm. Although this practice may be beneficial during the exploratory phase of VR paradigm development, failure to correct for multiple comparisons may provide misleading results whereby a correlation is found between two constructs incidentally. Conversely, some traditional tasks assessed multiple constructs, which poses a slightly different challenge. For example, if the VR paradigm broadly assessed EF but was validated against the ST, it was then unclear whether the VR paradigm aimed to assess processing speed, attention, inhibitory control, or interference control as the ST could be used to measure all four. Similarly, when these studies used multiple traditional assessments, the reader was expected to presume the target constructs of the VR paradigm as this was not clearly outlined. Poorly defined target constructs and failure to specify which traditional task validates which aspect of the VR task produces a literature that is difficult to interpret. Moreover, this general lack of clarity means that future researchers are more likely to invent a new paradigm rather than adopt or extend existing paradigms, creating inefficiency and hampering progress in the field.

Various standardized tasks were used to validate target constructs in the VR paradigm. For example, the study by Chicchi Giglioli et al [69] examined attention and inhibition control using the DPT, GNG, and ST. However, Voinescu et al [71] examined inhibition using the CPT paradigm. In addition, Marín-Morales et al [70] assessed inhibition using one mini-game of their VR paradigm. However, they neither provided details of a specific comparator task for validation purposes nor reported the statistical outcomes. Furthermore, the DPT, which is typically used to assess selective attention [88], was used to assess inhibition, although its own psychometric properties have been the subject of controversy [89,90]. Although several traditional tasks purport to measure the same construct (ie, there is not one task for one construct), the lack of consistency between studies makes it difficult to compare VR platforms. Furthermore, the traditional comparator task used to validate the VR paradigm needs to have sound psychometric properties in its own right to assess the respective construct; when two tasks are compared with one another, it is unclear which task may be responsible for discrepancies in the outcome [91]. These points are especially pertinent for studies that rely solely on traditional measures to validate tasks in the absence of other validation techniques.

Although it is promising to see that VR paradigms are being used for ecologically valid assessments, their validation remains a challenge. In the case of traditional tasks, we assume that a single construct can be assessed using a behavioral task and that the performance on that task is linear with the cognitive construct. In the case of a “function-led” VR task, there is a behavioral task that simulates real-world functioning, which is thought to deteriorate in an EF-declining population. This VR task is not a direct assessment of a target construct—it is a test of a real-world function, such as parking a car. To test convergent validity, the individual would have to park a car in real life and have their performance assessed similarly to that on the VR task and compared. However, when we use traditional measures to validate the “function-led” VR measures, we assume that EF can be reliably measured and the function-led VR task (eg, parking a car) requires the same EF. Thus, those who perform poorly on a traditional EF task are also expected to perform poorly on real-life tasks requiring EF. Critically, if our results do not show this relationship, it could be that the traditional task is a poor test of EF, the function-led assessment is a poor test of EF, or the EF at hand is not related to the functional task (eg, parking a car).

These assumptions place substantial weight on the selection of the traditional task for validating the VR paradigm for predictive validity. Davison et al [62] assessed EF using the ST and TMT. They broadly hypothesized that there would be correlations between the traditional measures and the VR paradigm, which contained tasks that replicated real life, such as car parking, arranging seating, and locating items. In the reported results, the ST and TMT were correlated with all outcome measures of the VR paradigm. For example, performance on the Stroop Color and Word Test was correlated with performance on the second parking simulator task, the number of levels completed on the parking simulator task, and the time taken to place the blue chair in the seating arrangement task. If the ST and TMT are not sufficient validators of the functional task, this may generate misleading results regarding the integrity of the VR paradigm and its ability to sensitively measure EF. Thus, the convergent validity of VR tasks would be better assessed through real-life performance on the same task, such as actually parking in a controlled environment. Although this may seem to be a resource burden to validation, it could provide integral merit to using the paradigm as a proxy for the real-life task thereafter. Alternative options are to assess convergent validity through other forms of real-life functioning (eg, self-care, residence, transportation, and employment) and diagnostic trajectory [49]. Moreover, predictive validators should be carefully chosen to ensure that their target construct aligns with that thought to be required for the function-led assessment.

Nonetheless, for novel task validation, transparent reporting of all results is crucial for advancing future research. Several papers included in this review (4/19, 21%) [61,62,68,69] reported only statistically significant correlations, leaving unanswered questions because of the omission of nonsignificant results. For instance, Chicchi Giglioli et al [69] sought to evaluate inhibition control using the GNG and ST for validation (both are common tasks for assessing inhibition) as well as the DPT yet did not report all correlational data in their results table. Such omissions hinder the comprehensive use or meta-analytic application of these findings. Conversely, Chicchi Giglioli et al [68] provided a detailed comparison between each validation task and its corresponding VR task, including the constructs assessed. However, only significant correlations were reported, some of which were between tasks intended to assess disparate constructs, such as the correlations between the Wisconsin Card Sorting Test (assessing cognitive shifting) and the VR tasks (measuring attention and inhibition control). Although these findings may indicate overlapping constructs in VR tasks, the absence of multiple-comparison correction and a detailed post hoc analysis of these correlations limits the interpretability and applicability of these results.

Finally, it is worth noting that there was significant variation in sample sizes across the studies reviewed. Although it is often accepted that pilot studies or preliminary studies have small sample sizes that often result in underpowered analysis, the utility of the VR paradigms is dependent on sound psychometric properties that require adequate sample sizes and statistical power. As detailed in Multimedia Appendix 1 [53-55,61-76], the sample sizes varied from 12 (6 per group) [74] to 103 (divided into 2 groups) [53]. Although the definition of a “sufficient” sample size may vary between studies and analyses, several of the included VR paradigms would likely require additional validation studies to provide confidence in their psychometric properties.

#### Recommendations

Our recommendations are as follows:

1. Papers should explicitly detail how their VR paradigms are being validated. If a paradigm has multiple components, it is essential to state how each one is being validated. A good example is the paper by Kourtesis et al [76] in this review.
2. If studies aim to validate a VR paradigm for a specific EF construct, they should identify a priori the precise outcome measures of the VR paradigm that are hypothesized to tap into various EF constructs (eg, time to completion and number of errors) and then validate them against the appropriate traditional tasks that also reliably assess those EF constructs.
3. Where appropriate, the VR paradigm’s real-world task should be validated against both traditional task measures and ecologically valid measures. Ecologically valid measures may include carer reports, observation assessments, and activity of daily living assessments.
4. Multiple modes of validation should be used, including measures that provide predictive power [49], and both carer reports of daily functioning and biosensor data should be considered.
5. Papers should report all outcomes of validation data (even those in supplementary materials) to ensure the transparency of the tools’ properties. A concerted effort to increase explicit and transparent reporting would greatly benefit this field.
6. To validate the VR paradigm, the psychometric properties of the traditional task must be appropriate.
7. Studies aiming to evaluate the psychometric properties of their VR paradigm should ensure that they have adequate sample sizes for a powered analysis.

### Cybersickness

#### Overview

Although VR offers several key advantages over traditional tasks, these systems can also produce adverse effects such as cybersickness. In our review, only 21% (4/19) of the studies included an assessment of cybersickness. This is concerning as cybersickness presents a substantial confound for valid VR assessment and has been shown to negatively affect task performance [92,93]. Given that the assessment of EF involves ascertaining a participant’s cognitive abilities, the recording of cybersickness is key to ensuring that common side effects such as dizziness and vertigo do not affect the participants’ ability to perform at their best on the tasks. Without formal evaluation, the degree to which participants’ experiences are altered is unclear. Furthermore, it is unknown at this stage whether cybersickness symptoms affect various client populations differently. For example, it is possible that, although a healthy individual may be able to continue the assessment with minor vertigo, an individual with cognitive impairment may be more affected, resulting in severely affected cognitive outcomes. Thus, caution should be exercised when using VR paradigms to ensure that the potential benefits of engagement and ecological validity are not realized at the cost of the potential negative effects of cybersickness.

#### Recommendations

Our recommendations are as follows:

1. Future papers should include usability data in the form of cybersickness measurements.
2. Correlations between cybersickness and participants’ task performance could be included as supplementary material that should be accessible to readers, enabling them to better understand how the VR battery is performing.
3. Even when a paradigm has already assessed cybersickness, we encourage future researchers to use the same paradigm to conduct their own cybersickness assessments. This is because it is still unclear whether cybersickness will have different effects on various populations.
4. Clinical researchers and engineers should continue to investigate and report on techniques and technologies that reduce the incidence or severity of cybersickness.

Textbox 1 provides an overview of the recommendations of this review.

**Recommendations for future research and practice using virtual reality (VR) head-mounted display–based paradigms for executive functioning (EF) assessment.**

**Validate against multiple forms**

- Examples include carer reports, observation assessments, ecological momentary assessments, activity of daily living assessments, physiological sensors, and in vivo studies.
- Consider longitudinal tracking of participants to establish predictive utility to initially validate the novel paradigm.

**Report a priori how each assessment in the VR paradigm is being validated**

- If there are multiple components to one paradigm, state how each element is being validated (a good example is the study by Kourtesis et al [76] in this review); for example: “Task 2a aims to assess inhibitory control and is validated against the traditional stop signal task and go/no-go task.”

**Report all validation data**

- Report correlations of all aspects of a task that were identified a priori as validating the VR paradigm. In extending the previous example, show all relevant metrics from task 2a, such as errors, proportion of successful stops, reaction time, and stop signal reaction time against the relevant metrics of both the Stop Signal and Go/No-Go tasks.

**Include user experience assessment**

- Conduct assessments of immersion, cybersickness, usability, and engagement.

**Use a common framework for defining target constructs**

- The Research Domain Criteria is one option of a framework that can be applied to ensure that terminology used in the field is consistent.

**Consider adding biosensors**

- These provide additional objective data that may inform the VR-based EF assessment.

### Limitations

We searched for articles that used the terms “executive functioning,” “higher order cognition,” and “functional assessment” to capture tasks that aimed to broadly assess facets of EF. This search strategy may have missed studies that examined a key construct of EF but did not specifically use the aforementioned terms (eg, used VR to assess inhibitory control alone). In addition, we did not contact the authors of the papers included in this review for further information; however, one of the key outcomes of this review was the amount of information contained in the manuscripts for future studies to extend upon.

### Future Directions

The authors posit that the integration of biosensors into a VR system has significant potential. Biosensors such as pupillometry, eye gaze, EEG, and language and grammatical characteristic data can be temporally linked to the events occurring in the VR task. For example, pupillometry can offer insights into brain injury prognosis [94] and differentiate between participants with Alzheimer disease and healthy controls [95]. Eye tracking during reading aids Alzheimer disease identification [96], and linguistic attributes (eg, formation and fluency of sentences, syntax, and grammar) distinguish patients with Alzheimer disease from those with MCI [97]. The combination of these biosensor metrics and real-time function-led VR performance could increase the sensitivity of tests, enabling the detection of subtle differences such as between MCI and subjective memory complaints [98]. However, currently, biosignals are rarely evaluated alongside emerging VR paradigms for EF assessment. None of the reviewed studies used biosensors, leaving an untapped potential for VR paradigms to be frontline neuropsychological assessments.

Biosensors could also assist in modulating the cognitive load experienced by participants. Cognitive load is the cumulative working memory resources that an individual requires for a given task [99]. Similar to the gaming industry, VR paradigms could be adaptive and performance driven so that the level of challenge adjusts according to real-time individual responses [100,101]. Modulating the cognitive load adjusts the challenge of a task and enables all participants to encounter similar levels of perceived difficulty for their respective abilities. EEG, pupillometry, and cardiovascular measures are also sensitive to cognitive load capacities [99].

An additional advantage of VR is its ability to facilitate the assessment of spatial navigation. Spatial navigation is a component of cognitive functioning that can be a key factor in detecting early stages of neurodegenerative diseases. However, it cannot be assessed adequately by means of many traditional assessments. Although it is acknowledged that spatial navigation is not a component of EF, the authors of this paper consider it a generally underexamined construct when assessing cognition and general function. For example, spatial navigation is a cognitive marker used to detect early attention deficit [102,103] and offers additional relevant information beyond the traditional neuropsychological tests [103]. The environment could also be systematically manipulated to match the needs of the assessment [104] and tailored to specific populations. However, typically, spatial navigation is assessed using a real-space human analog of the Morris water maze test, which can be difficult to implement under standardized conditions. Computerized versions have been adapted, with findings comparable with those of tests conducted in real space [105], suggesting promise for translating this style of assessment to VR.

### Conclusions

VR paradigms assessing EF have great potential to improve upon traditional tests. However, despite their undeniable novelty and potential, their methodological and psychometric properties must be addressed during their development to ensure their validity and reliability. Although there is no shortage of research in this area, the lack of standardized protocols to validate VR-based neuropsychological assessments hinders the progress of this field of research and practice. It is hoped that this study will be the beginning of a larger movement toward systematizing the development and validation of these paradigms.

## Appendix

Multimedia Appendix 1 Article details including participant demographics, virtual reality (VR) paradigm, VR tasks, measures of user experience, and comparative assessments for VR paradigms.

Multimedia Appendix 2 PRISMA checklist.

## Abbreviations

CAVIR: cognition assessment in virtual reality
CPT: continuous performance test
DPT: dot-probe task
EAL: Everyday Assessment Lab
EEG: electroencephalography
EF: executive functioning
FAST: Functioning Assessment Short Test
GNG: Go/No-Go
MCI: mild cognitive impairment
MET: Multiple Errands Test
PRISMA: Preferred Reporting Items for Systematic Reviews and Meta-Analyses
RDoC: Research Domain Criteria
ST: Stroop test
TMT: Trail-Making Test
TMT-A: Trail-Making Test version A
TMT-B: Trail-Making Test version B
UPSA-B: brief University of California, San Diego, Performance-Based Skills Assessment
VMT: Virtual Multitasking Test
VR: virtual reality
WoS: Web of Science

## Data Availability

All data generated or analyzed during this study are included in this published article (and its supplementary information files).
