# Supplementary material for: Immersive Virtual Reality–Based Methods for Assessing Executive Functioning: Systematic Review
Source: JMIR Serious Games. 2024 Feb 26;12:e50282. doi: 10.2196/50282 (PMC10928525; doi:10.2196/50282)
Supplement: Multimedia Appendix 1 [file games_v12i1e50282_app1.docx]

Table S1. Article details including participant demographics, VR paradigm, VR tasks, measures of user experience and comparative assessments for VR paradigms.

| Author and Year | Participants | | | Age | | | Virtual Environment | Virtual Task | User Experience Assessment | Comparative Assessment |
| --- | --- | --- | --- | --- | --- | --- | --- | --- | --- | --- |
|  | N | F | M | M | SD | Range |  |  |  |  |
| Banville et al. (2018) | Group 1 | | | Group 1 | | | Seven different rooms in virtual apartment | Sort groceries while distractors were presented | Discomfort SSQ  Presence IPQ | HTT, SET, ST, TMT, ZMT |
|  | 14 | 11 | 3 | 26.1 | 6.2 | 18-45 |  |  |  |  |
|  | Group 2 | | | Group 2 | | |  |  |  |  |
|  | 11 | 8 | 3 | 66.8 | 2.9 | 60-87 |  |  |  |  |
| Chicchi Giglioli, de Juan Ripoll et al. (2021) | Group 1 | | | Group 1 | | | A spaceship | 8 missions  Take off  Sort resources  Kill aliens  Repair valve  Source water  Grow plants  Find fuel  Lock room | N.D. | Computerized version:  DOT, GNG, ST, TMT, TOL-DX, WCST |
|  | 47 | 27 | 20 | 26.7 | 11.2 | N.D. |  |  |  |  |
|  | Group 2 | | | Group 2 | | |  |  |  |  |
|  | 47 | 30 | 17 | 31.6 | 8.8 | N.D. |  |  |  |  |
| Chicchi Giglioli, Pérez Gálvezet et al. (2021) | Group 1 | | | Group 1 | | | Kitchen | Cook a meal using various ingredients using one or two cookers | N.D. | DOT, GNG, ST, TMT, TOL-DX |
|  | 24 | 14 | 9 | 44.7 | 9.7 | N.D. |  |  |  |  |
|  | Group 2 | | | Group 2 | | |  |  |  |  |
|  | 18 | 11 | 7 | 45.4 | 9.8 | N.D. |  |  |  |  |
| Davison et al. (2017) | Group 1 | | | Group 1 | | | 1. Garage  2. Chemistry Lab | Park car in various parking spaces  Arrange chairs, locate items in lab | No formal assessment (e.g., SSQ/PQ) | ST, TMT |
|  | 22 | 10 | 12 | 20.6 | N.D. | 18-25 |  |  |  |  |
|  | Group 2 | | | Group 2 | | |  |  |  |  |
|  | 18 | 11 | 7 | 69.9 | N.D. | 65+ |  |  |  |  |
| Kourtesis et al. (2021) | 41 | 21 | 20 | 29.2 | 5.8 | N.D. | Bedroom, kitchen, living room, garden, in a car, supermarket, bakery, and library | Perform variety of tasks in 22 scenarios, such as cooking a meal or purchasing items from supermarket | Discomfort  VRNQ | BADS, CAMPROMPT, CTT, RSAT, TEA, TMT |
| Kourtesis & MacPherson (2021) | 41 | 21 | 20 | 29.2 | 5.8 | N.D. | Same as Kourtesis et al. (2021) | Same as Kourtesis et al. (2021) | Discomfort  VRNQ |  |
| Marin-Morales et al. (2021) | 60 | 30 | 30 | 36.0 | 11.2 | N.D. | Extra-terrestrial planet | 42 minigames, but only 4 disclosed | N.D. | DOT, GNG, ROCFT, ST, TMTB, TOLDX |
| Miskowiak et al. (2022) | Group 1 | | | Group 1 | | | Kitchen | Cook a dish | Discomfort  VRSSQ  Presence  PQ | CANTAB-RVP, OTS, RAVLT, RBANS, SWM |
|  | 40 | 26 | 14 | 30.4 | 9.6 | N.D. |  |  |  |  |
|  | Group 2 | | | Group 2 | | |  |  |  |  |
|  | 40 | 24 | 16 | 35.3 | 11.7 | N.D. |  |  |  |  |
|  | Group 3 | | | Group 3 | | |  |  |  |  |
|  | 41 | 17 | 23 | 24.2 | 3.9 | N.D. |  |  |  |  |
| Pallavicini et al. (2019) | 28 | 13 | 25 | 25.8 | 4.14 | N.D. | Horizon-like environment | Hit approaching orbs (dance-based game) | N.D. | TMT |
| Parsons & Carlew (2016) | *Study 1* | | | *Study 1* | | | Classroom | Perform Stroop task shown on blackboard | N.D. | ST (D-KEFS), ST (ANAM) |
|  | 50 | 39 | 11 | 20.4 | N.D. | 18-30 |  |  |  |  |
|  | *Study 2* | | | *Study 2* | | |  |  |  |  |
|  | Group 1 | | | Group 1 | | |  |  |  |  |
|  | 8 | N.D. | N.D. | 22.9 | N.D. | 18-34 |  |  |  |  |
|  | Group 2 | | | Group 2 | | |  |  |  |  |
|  | 10 | N.D. | N.D. | 18.8 | N.D. | 18-20 |  |  |  |  |
| Parsons & McMahan (2017) | *Study 1* | | | *Study 1* | | | Grocery Store | Deliver prescriptions  Buy groceries  Grab coupons | No formal assessment (e.g., SSQ/PQ) | CVLT–II,  ST (D-KEFS) |
|  | 42 | 27 | 15 | 19.8 | 2.8 | N.D. |  |  |  |  |
|  | *Study 2* | | | *Study 2* | | |  |  |  |  |
|  | 61 | 34 | 27 | 20.9 | 3.7 | N.D. |  |  |  |  |
| Parsons & Barnett (2019) | Group 1 | | | Group 1 | | | Living room of an apartment | Perform Stroop task on television | N.D. | ST (D-KEFS), ST (ANAM) |
|  | 39 | 29 | 10 | 74.4 | 8.7 | N.D. |  |  |  |  |
|  | Group 2 | | | Group 2 | | |  |  |  |  |
|  | 50 | 43 | 7 | 20.6 | 3.2 | N.D. |  |  |  |  |
| Porffy et al. (2021) |  |  |  |  |  |  | Store | Recall items on list  Find items on list  Pay for items on list  Purchase a coffee | N.D. | GMLT |
| Robitaille et al. (2017) | Group 1 | | | Group 1 | | | Courtyard | Respond to hostile and non-hostile faces in windows | Discomfort  SSQ, SUSQ  Presence  PQ | N.D. |
|  | 6 | N.D. | N.D. | N.D. | N.D. | N.D. |  |  |  |  |
|  | Group 2 | | | Group 2 | | |  |  |  |  |
|  | 6 | N.D. | N.D. | N.D. | N.D. | N.D. |  |  |  |  |
| Tan et al. (2022) | Group 1 | | | Group 1 | | | Virtual representation of various environments in Singapore | Brush teeth  Make sandwich  Read newspaper  Watch weather forecast  Remember shopping list  Choose clothes  Leave house  Cross street  Find fruit stall  Buy fruit | N.D. | N.D. |
|  | 24 | 12 | 12 | N.D. | N.D. | 35-44 |  |  |  |  |
|  | Group 2 | | | Group 2 | | |  |  |  |  |
|  | 25 | 14 | 11 | N.D. | N.D. | 45-54 |  |  |  |  |
|  | Group 3 | | | Group 3 | | |  |  |  |  |
|  | 25 | 13 | 12 | N.D. | N.D. | 55-64 |  |  |  |  |
|  | Group 4 | | | Group 4 | | |  |  |  |  |
|  | 25 | 16 | 9 | N.D. | N.D. | 65-74 |  |  |  |  |
| Tsai et al. (2021) | Group 1 | | | Group 1 | | | Supermarket | MET | N.D. | N.D. |
|  | 6 | 3 | 3 | 74.8 | 4.1 | N.D. |  |  |  |  |
|  | Group 2 | | | Group 2 | | |  |  |  |  |
|  | 6 | 3 | 3 | 70.2 | 5.2 | N.D. |  |  |  |  |
| Voinescu et al. (2021) | Group 1 | | | Group 1 | | | An aquarium | Respond to seeing a specific fish or hearing a specific word | Discomfort  SSQ  Presence  PQ | CBT, CPT, MoCA, ST, TMT |
|  | 41 | N.D. | N.D. | N.D. | N.D. | N.D. |  |  |  |  |
|  | Group 2 | | | Group 2 | | |  |  |  |  |
|  | 41 | N.D. | N.D. | N.D. | N.D. | N.D. |  |  |  |  |
| Wilf et al. (2022) | Group 1 | | | Group 1 | | | Purple environment with trail points spaced in 3D around participant | CTT | N.D. | CTT |
|  | 39 | 21 | 18 | 26.7 | 4 | N.D. |  |  |  |  |
|  | Group 2 | | | Group 2 | | |  |  |  |  |
|  | 50 | 38 | 12 | 56.1 | 6 | N.D. |  |  |  |  |
|  | Group 3 | | | Group 3 | | |  |  |  |  |
|  | 14 | 10 | 4 | 72.6 | 5 | N.D. |  |  |  |  |
| Williams et al. (2022) | 15 | 6 | 7 | 23 | N.D. | N.D. | Empty room where tasks appear | Wisconsin Sorting Task  Multi-tasking Task  Mixed Sorting Task | N.D. | AEFI, MT, WSCT |

Note: F = Female, M = Male, N = Number of participants, N.D. = Non-disclosed. Other abbreviations for comparative assessments: AEFI = Amsterdam Executive Function Inventory, BADS = Behavioural Assessment of the Dysexecutive Syndrome, CAMPROMPT = Cambridge Prospective Memory Test, CBT = Corsi Block Test, CPT = Continuous Performance Test, CVLT–II = California Verbal Learning Test—Second Edition, DOT = Dot Probe Task, GNG = Go No-Go Task, GLMT = Groton Maze Learning Task, HTT = Hanoi Tower Test, IPQ = Igroup Presence Questionnaire, MoCA = Montreal Cognitive Assessment, MT = Multitasking Task, OTS = One Touch Stocking of Cambridge , PQ = Presence Questionnaire, RAVLT = Rey Auditory Verbal Learning Test, RBANS = Repeatable Battery for the Assessment of Neuropsychological Status, ROCF = Rey-Osterrieth Complex Figure Test, RSAT = Ruff Selective Attention Test, SET = Six Element Test, SUS = System Usability Scale, SUSQ = Slater-Usoh-Steed Questionnaire, SSQ = Simulator Sickness Questionnaire, ST = Stroop Task, SWM = Spatial Working Memory, TEA = Test of Everyday Attention, TMT = Trail Making Test, TOL-DX = Tower of London-Drexler test, WCST = Wisconsin Card Sorting Test, ZMT = Zoo Map Test.
